# Automated clean-up, separation and detection of polycyclic aromatic hydrocarbons in particulate matter extracts using a 2D-LC/2D-GC system: a method translation from two FIDs to two MS detectors

**DOI:** 10.1007/s00216-017-0509-1

**Published:** 2017-07-24

**Authors:** Hwanmi Lim, Trifa M. Ahmed, Christoffer Bergvall, Roger Westerholm

**Affiliations:** 1 Department of Environmental Science and Analytical Chemistry (ACES), Stockholm University, 106 91 Stockholm, Sweden; 2 Livsmedelsverket, Box 622, 751 26 Uppsala, Sweden; 3 Department of Ecology and Genetics, Limnology, Uppsala University, 752 36 Uppsala, Sweden

**Keywords:** Polycyclic aromatic hydrocarbon, Multidimensional gas chromatography, Standard reference material, Wood smoke particulates, Long-term stability

## Abstract

An online two-dimensional (2D) liquid chromatography/2D gas chromatography system with two mass-selective detectors has been developed on the basis of a previous system with two flame ionization detectors. The method translation involved the change of carrier gas from hydrogen to helium, column dimension and detectors. The 2D system with two mass-selective detectors was validated with use of polycyclic aromatic hydrocarbon (PAH) standards and two standard reference materials from air and diesel exhaust. Furthermore, the system was applied to a real sample, wood smoke particulates. The PAH values determined correlated well with the previous data and those from the National Institute of Standards and Technology. The system enhanced the benefits of the previous system, which were limited by the low detectability and lack of mass selectivity. This study shows an automated 2D system that is valid for PAH analysis of complex environmental samples directly from crude extracts.

**Electronic supplementary material:**

The online version of this article (doi:10.1007/s00216-017-0509-1) contains supplementary material, which is available to authorized users.

**Graphical Abstract:**
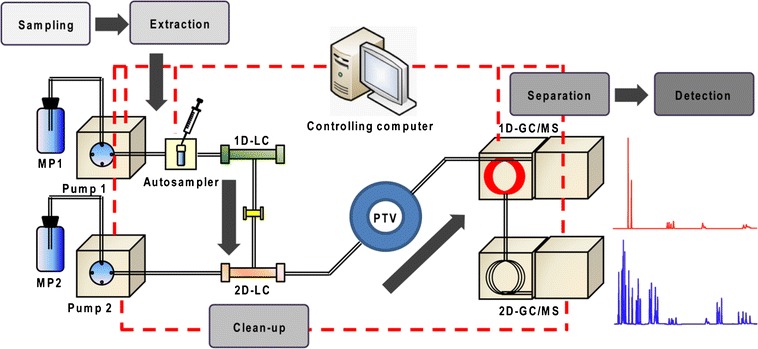
Schematic illustration showing on-line clean-up, separation and detection using 2D-LC/2D-GC/MS

## Introduction

Multidimensional chromatography (MDC) has been an alternative to single chromatography for analysis of environmental samples which may contain various compounds of interest as well as complex matrices [1]. Especially, multidimensional gas chromatography (MDGC) has had the main role because of its high separation capacity with the help of capillary gas chromatography (GC) columns [2]. A classic MDGC approach used so-called heart-cut via a flow switching device (GC–GC) located between the two columns to direct a fraction of the compounds from the first column to the second for further separation [3]. Another mode in MDGC is comprehensive GC × GC, which was introduced later, where a modulator continuously collects and transfers small effluents from the first column to the second column [4]. Both modes are complementary and intended to maximize the peak capacity [5]. GC–GC is more applicable for identifying and studying known compounds, whereas the latter mode is used more extensively for characterization and classification of known and unknown compounds [6, 7].

Polycyclic aromatic hydrocarbons (PAHs), a subgroup of polycyclic aromatic compounds [8], are ubiquitous organic compounds found in the environment. PAHs are formed during incomplete combustion of organic matter mainly from anthropogenic activities (e.g. fossil fuel combustion, biomass burning, cooking and tobacco smoking) [9]. They are known to be carcinogenic and reprotoxic and for causing cardiovascular disease in animal studies [9, 10]. The International Agency for Research on Cancer classified benzo[*a*]pyrene as carcinogenic to humans, being the only compound in group 1 among the PAHs [11, 12]. The International Agency for Research on Cancer recently added outdoor air pollution to group 1, reinforcing the health risk of PAHs as they constitute the outdoor air with other possible sources, including ozone, particulate matter, sulfur dioxide and carbon monoxide [13].

The environmental and health-perspective importance of PAHs has driven the development of analytical methods capable of separating a range of compounds from low to high molecular weight as well as isomers and alkylated homologues from different matrices [14, 15]. MDC-based PAH analysis was one of the choices as it could simplify the complexity of the samples. A liquid chromatography (LC)-based MDC method was developed to maximize the separation of the historical 16 PAHs listed by the US Environmental Protection Agency [16]. In detail, a fractionation based on normal-phase LC first separated the PAHs according to the number of aromatic rings, then the following reversed-phase LC separated each fraction, which was later developed into an online system [17]. The multidimensional LC (MDLC) technique, however, was limited in use to certification of standard reference materials (SRMs) and could not be used for routine analysis [15]. Another MDC approach using GC expanded the number of analytes with increased peak capacity [15]. Heart-cut GC–GC, more conventional MDGC, is suitable for medium to complex samples with 100–200 analytes, and a comparably novel technique, comprehensive GC × GC, fits well with samples with high complexity [6]. The selection of appropriate column phases plays a key role in the multidimensional chromatographic separation [15, 18–20]. Amino and polymeric C_18_ LC columns, for instance, became preferable for MDLC, with the first column used for clean-up and isolation of the PAH fraction of interest from complex samples and the second column used for selective separation of each fraction containing PAHs with analytical difficulties (e.g. isomers or alkyl substituents) [21]. In MDGC, various GC columns have also been used to improve the separation mainly with capillary columns; for example, 5% and 50% phenyl methylpolysiloxane phases and/or 50% liquid crystal polysiloxane (LC50) [14, 15]. Columns with a 50% phenyl phase or similar especially demonstrated improved separation for isomeric PAHs of chrysene/triphenylene and benzo[*b*]fluoranthene/benzo[*j*]fluoranthene than a 5% phenyl phase, being the column of choice in PAH analysis [15]. An LC50 column, on the other hand, has been used as the other dimension in MDGC because of its shape selectivity which is similar to that of polymeric C_18_ phases, especially useful for isomer separation [22–24]. The 50% phenyl and LC50 columns were used in the previous two-dimensional (2D) GC system as the first and second dimension respectively [25]. Meanwhile, a novel phase called a *nano stationary phase* was introduced in comprehensive GC × GC as the second dimension to maximize the chromatographic orthogonality [23, 24].

A 2D system combining MDLC and MDGC was developed in which 2D-LC performed the sample clean-up and the fractionation enriched with PAHs, followed by 2D-GC analysis using heart-cut GC–GC [25]. The system was also shown to be valid for analysis of PAHs from urban dust and diesel engine exhaust particulates [25]. The detector used, a flame ionization detector (FID), however, limited the benefits of the system because of low detectability and lack of mass selectivity. The present study mainly aimed at method translation, changing the detector from an FID to a mass-selective detector (MSD), consequently entailing the use of helium instead of hydrogen as the carrier gas. The 2D system with an MSD at each end of the GC columns was validated and further applied to three different environmental matrices from air, diesel and wood smoke particulates.

## Materials and methods

### Chemicals and solvents

All solvents (high-performance LC grade), including methanol, methyl *tert*-butyl ether (MTBE) and toluene, were purchased from Rathburn Chemicals (Walkerburn, UK). Anhydrous dodecane (purity 99% or greater) was obtained from Sigma-Aldrich (St Louis, MO, USA). Dibenzothiophene (99%) was purchased from Janssen Chimica (Beerse, Belgium). All the other PAHs, including deuterated internal standards (ISs), used in this study were described previously [25, 26]. A full list of the PAHs and the ISs is presented in the electronic supplementary material, including the name, CAS Registry Number, and abbreviation (Table S1).

### Particulate samples

The same SRMs, SRM 1649a (urban dust) and SRM 1975 (diesel particulate extract) from the National Institute of Standards and Technology (NIST; Gaithersburg, MD, USA) were used as in a previous study [25]. Additionally, wood smoke particulates were collected from combustion of birch and fir wood in a small cast-iron stove situated in an exposure chamber [27].

### Sample preparation

SRM 1649a and SRM 1975 were prepared as described previously [25]. Briefly, 167 mg of SRM 1649a and one ampoule of SRM 1975 were placed on glass fibre filters (GF/C 47 mm, Whatman, Maidstone, UK) and spiked with the ISs separately. Then, the filter was inserted into a 5-mL extraction cell for pressurized liquid extraction (ASE 200 accelerated solvent extraction system, Dionex, Sunnyvale, CA, USA) using 9:1 (v/v) toluene/methanol as the extraction solvent at 20.7 MPa and 200 °C for 30 min and with five static cycles [28]. A blank filter was prepared in the same manner. Additionally, 3.6 mg of wood smoke particulates was weighed and prepared as well. Approximately, 90% of the whole extract was used for the previous study using 2D-LC/2D-GC/flame ionization detection [25], and the remaining amount was used for the LC–GC/mass spectrometry (MS) analysis and the present study. All the extracts used for the present study were stored in a freezer at -18 °C until analysis. The storage period of the crude extracts was nearly 4 years.

### Instrumentation

All the instrumentation for the 2D-LC/2D-GC system was described in the previous study [25]. The 2D-LC system was composed of three LC columns: C_18,_ 250 mm × 4.6 mm, 5 μm (Phenomenex, Torrance, CA, USA); Cosmosil 5-pentabromobenzyloxypropyl, 150 mm × 4.6 mm, 5 μm (Nacalai Tesque, Kyoto, Japan); and Hypercarb porous graphitic carbon (PGC), 10 mm × 4.6 mm, 3 μm (Thermo Fisher Scientific, Waltham, MA, USA). The 2D-GC system consisted of a 50% phenyl methylpolysiloxane column [low thermal mass (LTM) column module DB-17 ms], 30 m × 0.25 mm, 0.25-μm phase (Agilent Technologies, Folsom, CA, USA), and an LC50 column, 5 m × 0.25 mm, 0.10-μm phase (J&K Scientific, Milton, Canada). In the present study, the first dimension of the 2D-GC system was replaced with a shorter and thinner column (15 m × 0.25 mm, 0.15 μm; LTM DB-17 ms, Agilent Technologies, Folsom, CA, USA). Also, the detection method was modified by our changing the detectors from FIDs to MSDs. This was done by attachment of an additional MSD, a Finnigan TSQ 7000 triple-quadrupole mass spectrometer (Thermo Fisher Scientific, Waltham, MA, USA), onto the 7890A GC/5975C MSD (Agilent Technologies, Palo Alto, CA, USA) as shown in Fig. S1.

### Analysis by 2D-LC/2D-GC/MS

The automated clean-up, separation and detection scheme was illustrated previously [25]. In brief, 50 μL of IS-spiked extract was injected and passed through the first column (C_18_) with the mobile phase of methanol (1 mL/min). After the polar impurities had been sent to the waste, the flow was reversed to the second column (porous graphitic carbon) for peak focusing and mobile phase exchange. Before elution of the compound corresponding to the peak from the PGC column, the column flow was reversed again, and then directed to the third column (5-pentabromobenzyloxypropyl) with the mobile phase of MTBE (1 mL/min). The aliphatics and small aromatic PAHs (mono-PAHs and di-PAHs) were eluted earlier and sent to the waste. By reversal of the flow again, the PAH fraction was eluted as one backflush peak in the chromatogram and collected in the 500-μL injection loop of the programmable temperature vaporizer inlet of the GC/MS system via a transfer line (high-temperature deactivated fused-silica tubing). Then, large-volume injection was performed in solvent vent mode with a lowered flow rate (0.3 mL/min) of MTBE. The oven was kept at 50 °C until the injection had finished. Then, the sample went through the 2D-GC/MS system with use of heart cutting with the Deans switch valve programme. Details of the GC/MS parameters and valve programme are presented in Tables S2 and S3.

## Results and discussion

### GC method translation

The different parameters used for method translation are compared in Table 1. The initial method translation was done with Agilent GC method translation software (http://www.agilent.com/en-us/support/gas-chromatography/gcmethodtranslation) in “Translate” mode, and then modified to improve the resolution. Two different methods were investigated in this study. The first method (method 1) used a 20-m-long column as the second dimension to compensate for the decreased column efficiency due to the use of helium as the carrier gas. The other method (method 2) used a shorter first-dimension column (15 m) with higher phase ratio and with the second-dimension column kept short. The resolution gain from the longer column in method 1 was insufficient to overcome the low resolution from the first column. Method 2, instead, was chosen for further method development because of better separation in the first dimension. The total run time was 105 min, including the 20 min required for the sample injection and clean-up in the 2D-LC, whereas the previous system required 50 min as described previously [25]. This can be explained by the combined effect of the use of helium gas and inclusion of the late eluted dibenzopyrenes in this study.

**Table 1:** Comparison of method translation parameters between the previous and current two-dimensional (*2D*) gas chromatography (*GC*) systems

Parameters	Previous^a^	Method 1	Method 2	Reference^b^
System	2D-LC/2D-GC/FID	2D-LC/2D-GC/MS	2D-LC/2D-GC/MS	LC–GC/MS
Column outlet pressure	Atmospheric	Vacuum	Vacuum	Vacuum
Carrier gas	Hydrogen	Helium	Helium	Helium
Carrier gas flow	Pressure programme, 20–25 psi	Constant flow, 1 mL/min	Constant flow, 1 mL/min	Constant flow, 1 mL/min
Column dimension	1st: DB-17 ms, 30 m × 0.25 mm, 0.25 μm 2nd: LC50, 5 m × 0.25 mm, 0.10 μm	1st: DB-17 ms, 30 m × 0.25 mm, 0.25 μm 2nd: LC50, 20 m × 0.25 mm, 0.10 μm	1st: DB-17 ms, 15 m × 0.25 mm, 0.15 μm 2nd: LC50, 5 m × 0.25 mm, 0.10 μm	1st: DB-17 ms, 60 m × 0.25 mm, 0.15 μm
Phase ratio	1st: 250, 2nd: 625	1st: 250, 2nd: 625	1st: 417, 2nd: 625	1st: 417

*FID* flame ionization detection, *LC* liquid chromatography, *LC50* 50% liquid crystal polysiloxane, *MS* mass spectrometry

^a^Ahmed et al. [25]

^b^Sadiktsis et al. [26]

**Fig. 1:**
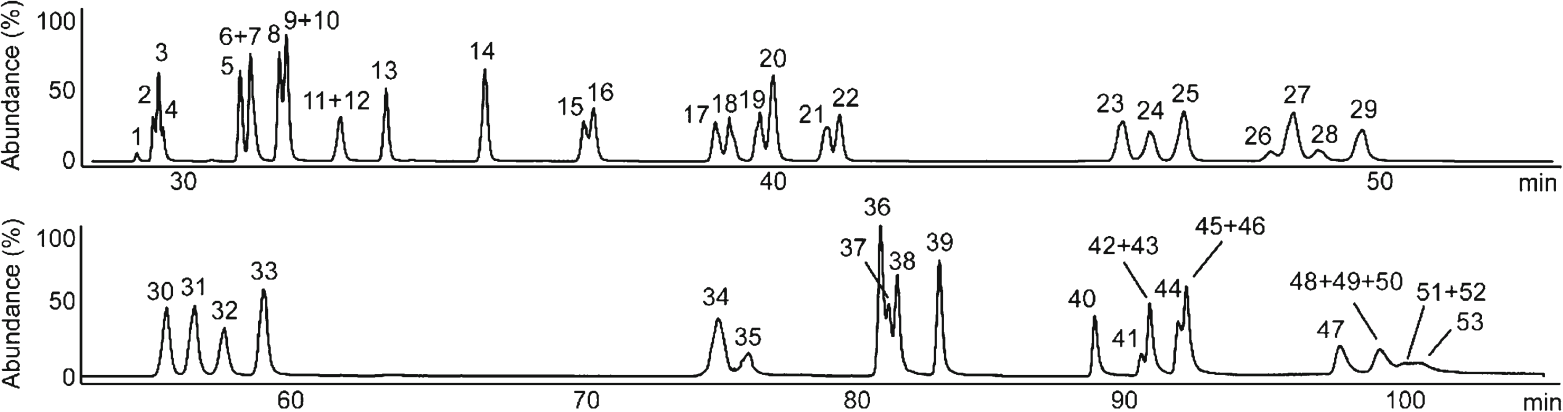
One-dimensional gas chromatography(GC)/mass spectrometry (MS) chromatogram obtained in selected-ion monitoring (SIM) mode with method 2. *1* DBT, *2* Phe-*d*_10_, *3* Phe, *4* Ant, *5* 3-MPhe, *6* 2-MPhe, *7* 2-MAnt, *8* 9-MPhe, *9* 1-MPhe, *10* 4*H*-CPP, *11* 9-MAnt, *12* 3,6-DMPhe, *13* 3,9-DMPhe, *14* Flu, *15* Pyr-*d*_10_, *16* Pyr, *17* 1-MFlu, *18* B[*a*]f, *19* B[*b*]f, *20* 2-MPyr, *21* 4-MPyr, *22* 1-MPyr, *23* B[*ghi*]F, *24* B[*c*]Phe, *25* B[*b*]NT, *26* B[*a*]A-*d*_12_, *27* B[*a*]A, *28* CPP, *29* Chr, *30* 3-MChr, *31* 2-MChr, *32* 6-MChr, *33* 1-MChr, *34* B[*b*]F, *35* B[*k*]F, *36* B[*e*]P, *37* B[*a*]P-*d*_12_, *38* B[*a*]P, *39* Per, *40* I[1,2,3-*cd*]F, *41* unknown, *42* I[1,2,3-*cd*]P, *43* DB[*a*,*h*]A, *44* Pic, *45* B[*ghi*]p-*d*_12_, *46* B[*ghi*]p, *47* DB[*a*,*l*]P, *48* Cor-*d*_12_, *49* Cor, *50* DB[*a*,*e*]P, *51* DB[*a*,*i*]P-*d*_14_, *52* DB[*a*,*i*]P, *53* DB[*a*,*h*]P. See Table 2 for an explanation of the abbreviations

**Fig. 2:**
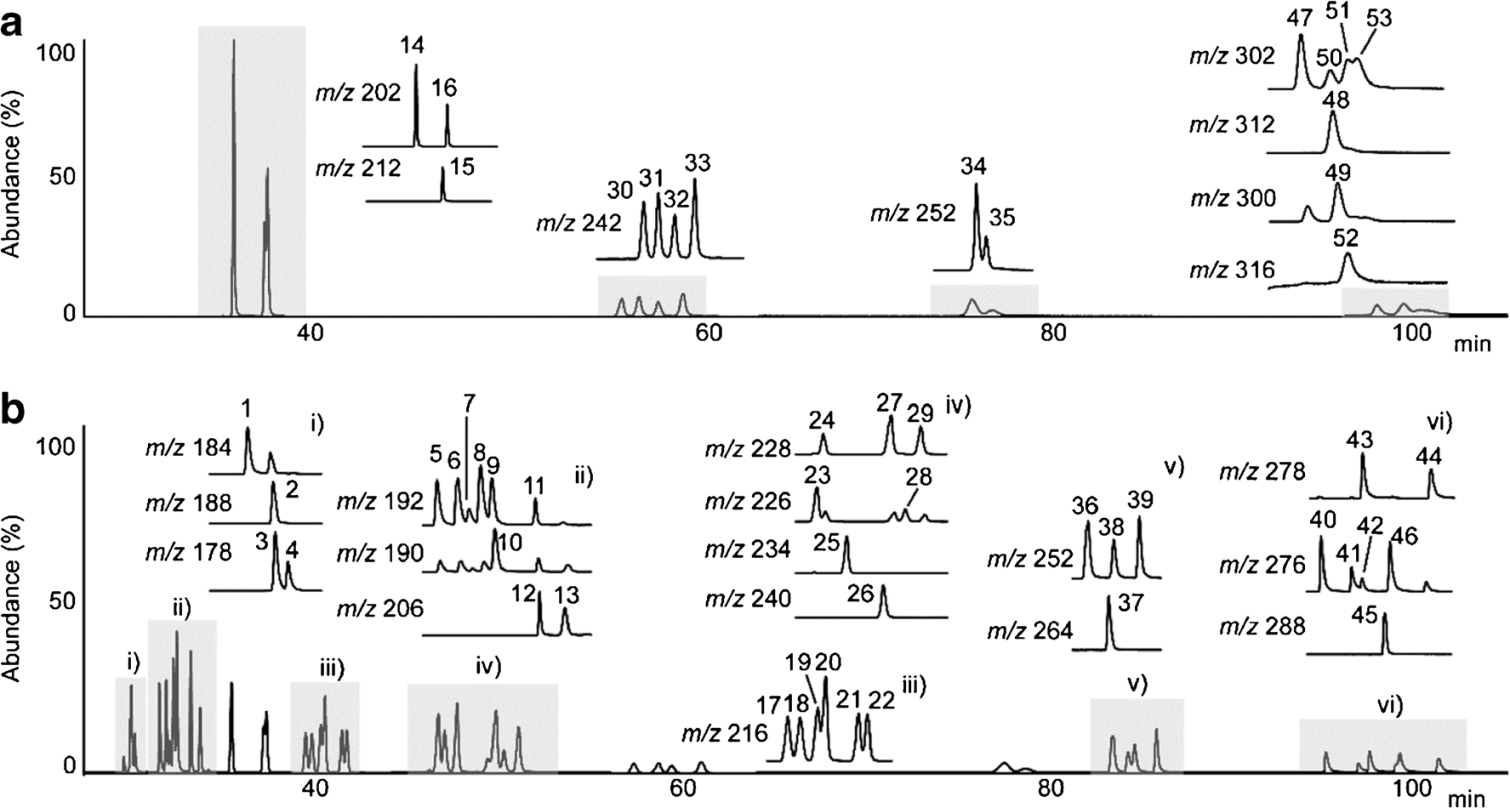
Two-dimensional (2D) GC/MS chromatogram obtained in SIM mode in the first dimension (**a**) and second dimension (**b**) with method 2 showing polycyclic aromatic hydrocarbon (PAH) isomer separation in the enlarged extracted ion chromatograms. *1* DBT, *2* Phe-*d*_10_, *3* Phe, *4* Ant, *5* 3-MPhe, *6* 2-MPhe, *7* 2-MAnt, *8* 9-MPhe, *9* 1-MPhe, *10* 4*H*-CPP, *11* 9-MAnt, *12* 3,6-DMPhe, *13* 3,9-DMPhe, *14* Flu, *15* Pyr-*d*_10_, *16* Pyr, *17* 1-MFlu, *18* B[*a*]f, *19* B[*b*]f, *20* 2-MPyr, *21* 4-MPyr, *22* 1-MPyr, *23* B[*ghi*]F, *24* B[*c*]Phe, *25* B[*b*]NT, *26* B[*a*]A-*d*_12_, *27* B[*a*]A, *28* CPP, *29* Chr, *30* 3-MChr, *31* 2-MChr, *32* 6-MChr, *33* 1-MChr, *34* B[*b*]F, *35* B[*k*]F, *36* B[*e*]P, *37* B[*a*]P-*d*_12_, *38* B[*a*]P, *39* Per, *40* I[1,2,3-*cd*]F, *41* unknown, *42* I[1,2,3-*cd*]P, *43* DB[*a*,*h*]A, *44* Pic, *45* B[*ghi*]p-*d*_12_, *46* B[*ghi*]p, *47* DB[*a*,*l*]P, *48* Cor-*d*_12_, *49* Cor, *50* DB[*a*,*e*]P, *51* DB[*a*,*i*]P-*d*_14_, *52* DB[*a*,*i*]P, *53* DB[*a*,*h*]P. See Table 2 for an explanation of the abbreviations

Method 2 had much better peak separation in the first dimension than method 1, especially for PAHs with *m*/*z* 216 (1-methylfluoranthene and 11*H*-benzo[*a*]fluorene) and *m*/*z* 252 (benzo[*b*]fluoranthene and benzo[*k*]fluoranthene) as shown in Fig. 1. All 53 PAH standards, including seven deuterated ISs, were separated as shown in the GC/MS chromatogram (Fig. 2). Of six unresolved peaks from the previous 2D system (peaks corresponding to phenanthrene-*d*_10_/phenanthrene, 9-methylphenanthrene/1-methylphenanthrene, pyrene-*d*_10_/pyrene, benzo[*c*]phenanthrene/benzo[*ghi*]fluoranthene, 6-methylchrysene/2-methylchrysene and benzo[*a*]pyrene-*d*_12_/benzo[*a*]pyrene) [25], the three PAH/IS pairs and benzo[*c*]phenanthrene/benzo[*ghi*]fluoranthene could be easily resolved by the MSDs. The remaining two pairs of PAH isomers (with *m*/*z* 192 and 242) were well separated with the current 2D system as shown in Fig. 2. Benzo[*b*]fluoranthene and benzo[*k*]fluoranthene, however, were partially co-eluted despite the use of the first column with a higher phase ratio. A four times longer column (60 m) was even unable to fully resolve those PAHs [26]. In addition, the attempt to separate them in the second dimension changed the elution order and created another co-elution problem, as shown previously [14]. The second column had better resolution for the four PAH isomers with *m*/*z* 216, but the separation between 11*H*-benzo[*b*]fluorene and 2-methylpyrene decreased as seen in Figs. 1 and 2b. Dibenzopyrenes were analysed on the first column because of peak broadening and the extended run time despite the partial co-elution (*m*/*z* 302 in Fig. 2a).

### Validation: linearity, limit of detection and limit of quantification

A series of calibration standards were prepared in triplicate and injected into the 2D system. The calibration curves were obtained by our plotting the peak area against the concentration. Calibration curves with seven concentrations were obtained for 43 PAHs, whereas for ten PAHs six levels were used and for seven PAHs five levels were used because of the low detectability at lower concentrations. The partially co-eluted dibenzo[*a*,*i*]pyrene and dibenzo[*a*,*h*]pyrene were determined together as shown in Table 2. The coefficient of determination (*R* ^2^) ranged from 0.987 to 0.998. The limits of detection and the limits of quantification (LOQs) were determined by injection of standard solutions at signal-to-noise ratios of 3 and 10 respectively.

**Table 2:** Summary of the linear range, correlation coefficient, limit of detection (*LOD*) and limit of quantification (*LOQ*)

PAH	Abbreviation	*m*/*z*	Linear range (pg)	*R* ^2^	LOD (pg)	LOQ (pg)
Dibenzothiophene	DBT	184	78.3–7830	0.992	19.6	78.3
Phenanthrene	Phe	178	227–22,700	0.993	56.8	227
Anthracene	Ant	178	79.0–7900	0.994	19.7	79.0
3-Methylphenanthrene	3-MPhe	192	218–21,800	0.991	54.5	218
2-Methylphenanthrene	2-MPhe	192	257–25,700	0.993	64.4	257
2-Methylanthracene	2-MAnt	192	81.2–8120	0.991	20.3	81.2
9-Methylphenanthrene	9-MPhe	192	282–28,200	0.997	70.6	282
1-Methylphenanthrene	1-MPhe	192	202–20,200	0.994	50.5	202
4*H*-Cyclopenta[*def*]phenanthrene	4*H*-CPP	190	148–14,800	0.994	37.1	148
3,6-Dimethylphenanthrene	3,6-DMPhe	206	134–13,400	0.996	33.5	134
9-Methylanthracene	9-MAnt	192	148–14,800	0.990	37.0	148
3,9-Dimethylphenanthrene	3,9-DMPhe	206	192–19,200	0.996	48.0	192
Fluoranthene	Flu	202	862–27,600	0.997	2.15	8.62
Pyrene	Pyr	202	453–14500	0.996	1.13	4.53
1-Methylfluoranthene	1-MFlu	216	185–18,500	0.995	46.2	185
11*H*-Benzo[*a*]fluorene	B[*a*]f	216	246–24,600	0.993	61.6	246
11*H*-Benzo[*b*]fluorene	B[*b*]f	216	277–27,700	0.994	69.4	277
2-Methylpyrene	2-MPyr	216	261–26,100	0.997	32.6	65.2
4-Methylpyrene	4-MPyr	216	145–14,500	0.994	36.3	145
1-Methylpyrene	1-MPyr	216	170–17,000	0.995	42.6	170
Benzo[*c*]phenanthrene^a^	B[*c*]Phe	228	316–12,600	0.996	126	316
Benzo[*ghi*]fluoranthene	B[*ghi*]F	226	184–18,400	0.997	45.9	184
Benzo[*b*]naphtho[1,2-*d*]thiophene	B[*b*]NT	234	127–12,700	0.998	31.7	127
Benz[*a*]anthracene	B[*a*]A	228	273–27,300	0.995	68.3	273
Cyclopenta[*cd*]pyrene^a^	CPP	226	380–15,200	0.992	152	380
Chrysene^a^	Chr	228	443–17,700	0.995	177	443
3-Methylchrysene	3-MChr	242	500–16,000	0.997	20.0	160
2-Methylchrysene	2-MChr	242	595–19,000	0.997	47.6	190
6-Methylchrysene	6-MChr	242	432–13,800	0.996	34.6	138
1-Methylchrysene	1-MChr	242	664–21,200	0.998	53.1	212
Benzo[*b*]fluoranthene	B[*b*]F	252	804–25,700	0.994	257	644
Benzo[*k*]fluoranthene	B[*k*]F	252	366–11,700	0.996	117	292
Benzo[*e*]pyrene	B[*e*]P	252	275–27,500	0.991	68.7	275
Benzo[*a*]pyrene^a^	B[*a*]P	252	523–20,900	0.995	209	523
Perylene^a^	Per	252	680–27,200	0.996	272	680
Indeno[1,2,3-*cd*]fluoranthene^b^	I[1,2,3-*cd*]F	276	1180–23,600	0.990	591	1180
Indeno[1,2,3-*cd*]pyrene^a^	I[1,2,3-*cd*]P	276	786–12,600	0.987	314	786
Dibenz[*a*,*h*]anthracene^b^	DB[*a*,*h*]A	278	1280–25,600	0.989	640	1280
Benzo[*ghi*]perylrene^b^	B[*ghi*]p	276	1050–21,000	0.990	526	1050
Picene^a^	Pic	278	1540–24,700	0.989	617	1230
Dibenzo[*a*,*l*]pyrene^a^	DB[*a*,*l*]P	302	1500–23,900	0.997	748	1500
Dibenzo[*a*,*e*]pyrene^b^	DB[*a*,*e*]P	302	1420–11,300	0.996	354	1420
Coronene^a^	Cor	300	1840–29,400	0.995	920	1840
Dibenzo[*a*,*i*]pyrene + dibenzo[*a*,*h*]pyrene^b^	DB[*a*,*i*]P + DB[*a*,*h*]P	302	3490–41,900	0.988	1750	3490
Internal standards						
Phenanthrene-*d*_10_	Phe-*d*_10_	188	213–21,300	0.990	53.3	213
Pyrene-*d*_10_	Pyr-d_10_	212	432–13,800	0.998	8.65	34.6
Benz[*a*]anthracene-*d*_12_^b^	B[*a*]A-*d*_12_	240	518–10,400	0.992	104	518
Benzo[*a*]pyrene-*d*_12_^a^	B[*a*]P-*d*_12_	264	534–21,400	0.994	214	534
Benzo[*ghi*]perylrene-*d*_12_^b^	B[*ghi*]p-*d*_12_	288	521–10,400	0.993	261	521
Coronene-*d*_12_	Cor-*d*_12_	300	654–10,500	0.997	262	523

PAH polycyclic aromatic hydrocarbon

^a^Based on the six-level calibration curve

^b^Based on the five-level calibration curve

The LOQs, in general, increased with retention times mainly from the peak broadening. As shown in Fig. S2, the trend was noticeable from indeno[1,2,3-*cd*]fluoranthene, and the LOQ of the last eluted dibenzo[*a*,*i*]pyrene and dibenzo[*a*,*h*]pyrene was nearly five times the average value for all PAHs. Apart from the additive effect of both columns on the peak broadening, an increasing temperature gap between the first-column and second-column temperature programmes (final temperature of 320 and 260 °C respectively) contributed to this trend when the compound from the first column started to be eluted at a temperature exceeding 260 °C, the final temperature of the second column. The temperature limit was the main downside of the LC50 column despite its unique selectivity for isomers. Alternative columns with a higher temperature limit as well as different selectivity have been introduced recently [23, 29]. A nano stationary phase (approximately 360 °C) instead of a 50% phenyl phase (Rxi-17) showed enhanced resolving power for PAH mixtures when combined with LC50 owing to their orthogonality [23, 24]. In addition, an ionic liquid phase is available for GC analysis with higher selectivity, stability and temperature above 350 °C [30]. Studies using ionic liquid phases for PAH analysis are limited, but are steadily on the increase in the attempt to utilize the dual nature retention selectivity [29, 31, 32]. The introduction of a novel stationary phase with a high temperature limit might advance the present method by decreasing LOQs for late eluted PAHs and further improving the resolution of isomeric PAHs.

### Sample analysis

**Fig. 3:**
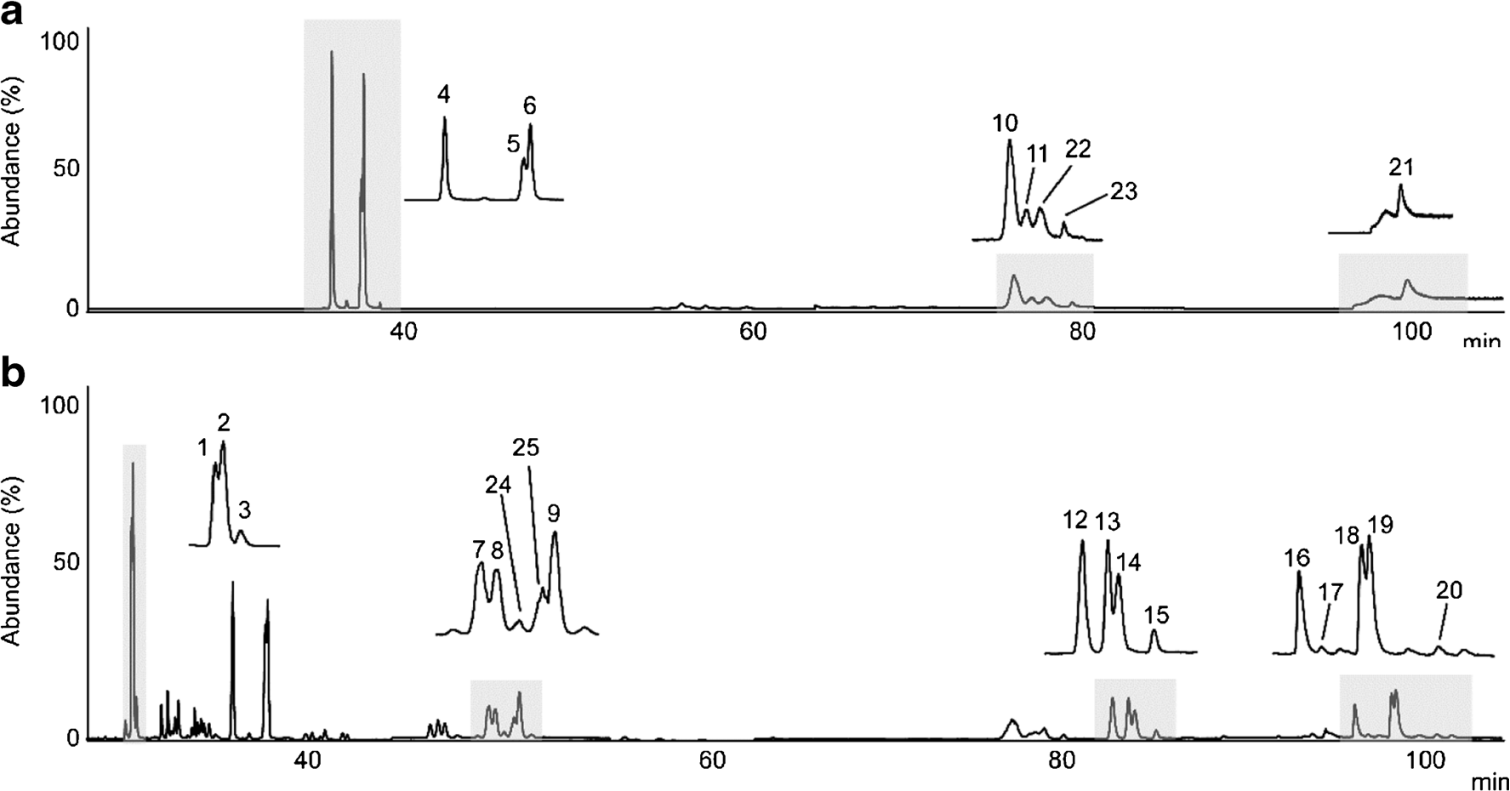
Two-dimensional GC/MS chromatogram obtained in SIM mode in the first dimension (**a**) and second dimension (**b**) obtained from urban dust (SRM 1649a). *1* Phe-*d*_10_, *2* Phe, *3* Ant, *4* Flu, *5* Pyr-*d*_10_, *6* Pyr, *7* B[*a*]A-*d*_12_, *8* B[*a*]A, *9* Chr, *10* B[*b*]F, *11* B[*k*]F, *12* B[*e*]P, *13* B[*a*]P-*d*_12_, *14* B[*a*]P, *15* Per, *16* I[1,2,3-*cd*]P, *17* DB[*a*,*h*]A, *18* B[*ghi*]p-*d*_12_, *19* B[*ghi*]p, *20* Pic, *21* DB[*a*,*e*]P, *22* benzo[*j*]fluoranthene, *23* benzo[*a*]fluoranthene, *24* CPP, *25* triphenylene. See Table 2 for an explanation of the abbreviations

Two SRMs, urban dust (SRM 1649a) and diesel particulate extract (SRM 1975), were used to validate the method developed. Furthermore, the 2D system was applied to a real sample, wood smoke particulates. All the samples were prepared at the same time as those used in the previous study [25] to minimize the variation in the sample preparation and examine the long-term storage stability of the crude extract. A representative chromatogram for SRM 1649a is presented in Fig. 3. The problematic PAH isomers with *m*/*z* 226 (cyclopenta[*cd*]pyrene), *m*/*z* 228 (benz[*a*]anthracene, chrysene and triphenylene) and *m*/*z* 252 (benzo[*b*]fluoranthene, benzo[*k*]fluoranthene, benzo[*j*]fluoranthene and benzo[*a*]fluoranthene) could not be separated, especially triphenylene and chrysene because they are normally co-eluted on the DB17-ms column [14].

#### SRM 1649a (urban dust) and SRM 1975 (diesel particulate extract)

**Fig. 4:**
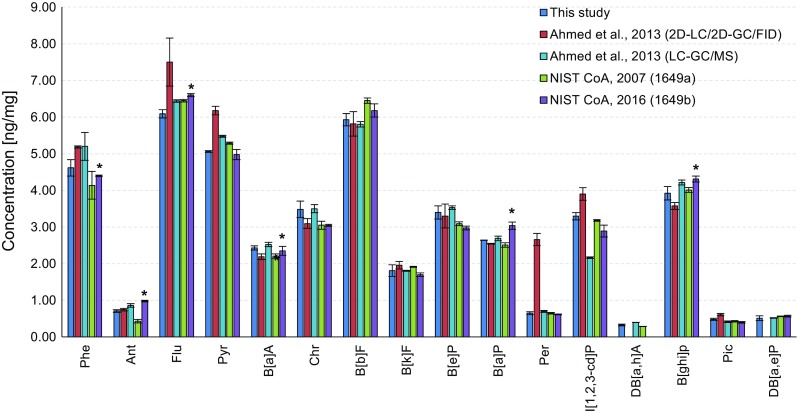
Comparison of results for PAH determination in SRM 1649a (urban dust) from the present study and the previous data determined by 2D liquid chromatography (LC)/2D-GC/flame ionization detection (FID) [25] and LC–GC/MS followed by solid-phase extraction (SPE) [25] and National Institute of Standards and Technology (*NIST*) certified values [33, 34]. See Table 2 for an explanation of the abbreviations. *Asterisks* reference mass fraction values based on pressurized fluid extraction at 200 °C

The PAH concentrations of SRM 1649a (urban dust) from this study were compared with those from the previous study together with NIST certified values as seen in Fig. 4. Most of the concentrations were within 20% or slightly higher (21% for picene) of the previous values except for anthracene, perylene and indeno[1,2,3-*cd*]pyrene. In the case of anthracene, the value was in good agreement with that obtained with the previous system but 64% higher than that from the NIST. The overestimation, however, was largely because of different extraction methods: the present study used pressurized liquid extraction and the NIST used classic Soxhlet extraction [33]. The difference was -28% when compared with the reference value determined with pressurized liquid extraction at 200 °C, strengthening the difference from the applied extraction method [34]. Although the lower chemical stability of anthracene mostly contributed to the underestimation [35], the problem of the abnormally higher value for perylene obtained with the previous system due to interference could be solved by the introduction of the MSD in the present study. The concentration of indeno[1,2,3-*cd*]pyrene determined in this study correlated well with the concentrations obtained with the previous 2D system and from the NIST, but not with the concentration obtained with the LC–GC/MS method [25].

**Fig. 5:**
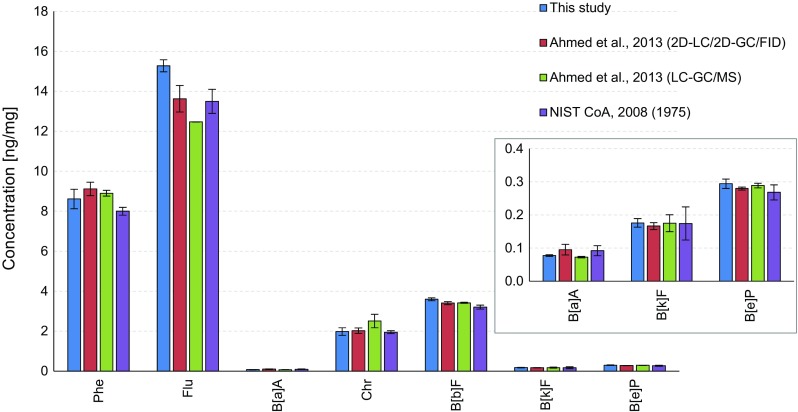
Comparison of results for PAH determination in SRM 1975 (diesel particulate extract) from the present study and previous data determined by 2D-LC/2D-GC/FID [25] and LC–GC/MS followed by SPE [25] and NIST certified values [36]. See Table 2 for an explanation of the abbreviations

The present 2D system was also validated for diesel particulate extract (SRM 1975). As seen in Fig. 5, the PAH concentrations determined were within 20% of the literature values, and only two values were just above 20% when compared with those obtained by LC–GC/MS [25, 36]. In detail, the concentrations of fluoranthene and chrysene were within 23% and -21% of the literature values, among which the slight underestimation of chrysene was owing to better separation between triphenylene and chrysene. However, their concentrations were in good agreement with those obtained with the previous 2D system and from the NIST.

#### Wood smoke particulate

**Fig. 6:**
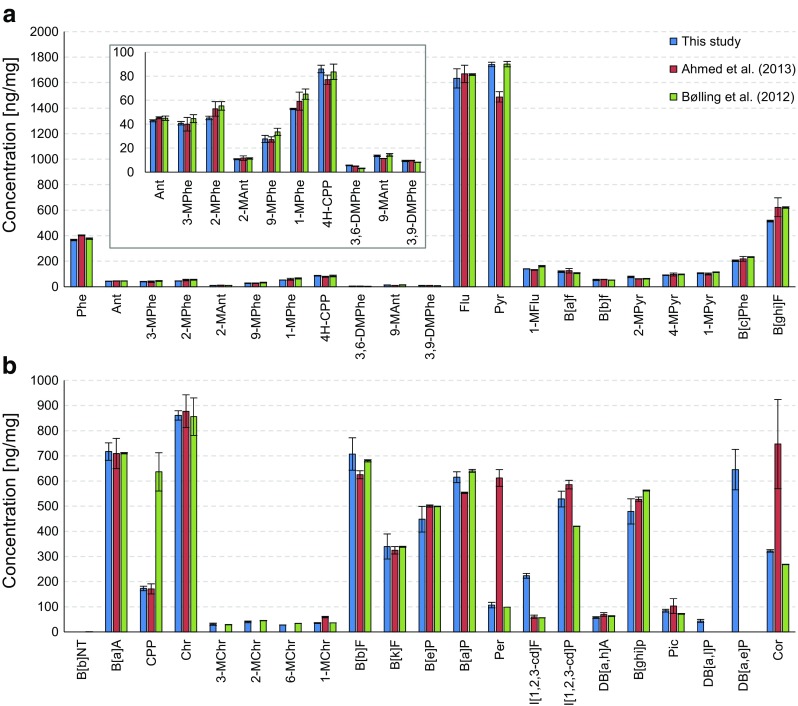
Comparison of results for the PAH determination in the wood smoke particulate sample from the present study and the previous data determined by 2D-LC/2D-GC/FID [25] and LC–GC/MS followed by SPE [27] from phenanthrene (*Phe*) to benzo[*ghi*]fluoranthene (*B*[*ghi*]*F*) (**a**) and from benzo[*b*]naphtho[1,2-*d*]thiophene (*B*[*b*]*NT*) to coronene (*Cor*) (**b**). See Table 2 for an explanation of the abbreviations

As shown in Fig. 6, the PAH concentrations in the wood smoke particulate sample were compared with those obtained with the previous system and the LC–GC/MS method [25, 27]. The concentrations were generally within 20% of the previous values, but the concentrations for 2-methylpyrene and indeno[1,2,3-*cd*]pyrene were within 26%. The determined concentrations of 1-methylchrysene, perylene and coronene were lower than those obtained with the previous 2D system, which was mainly caused by the difference in the detectors, indicating better selectivity of the MSD compared with the FID. Underestimation of another PAH, cyclopenta[*cd*]pyrene, when compared with the concentration determined by LC–GC/MS, on the other hand, was the result of improved separation of the 2D system regardless of the detectors. The overestimation of 3,6-dimethylphenanthrene with both 2D systems, however, was due to lower column efficiency than in the LC–GC/MS method, where a 60-m column was used. The concentration of indeno[1,2,3-*cd*]fluoranthene was abnormally high (approximately 300%) in the present study compared with previous studies. The sample matrix under long-term storage was thought to affect the stability of the fluoranthene-based compound, implying the determination of indeno[1,2,3-*cd*]fluoranthene should be done with care when it is stored for a long period.

In summary, the present 2D system was considered to be valid for PAH determination from three different matrices: urban dust, diesel particulate matter extract and wood smoke particulates. The determined concentrations, in general, agreed better with those obtained with the previous 2D system and from the NIST than those obtained by LC–GC/MS. Additionally, the long-term storage of the crude extract was mostly acceptable when compared with the previous results. However, anthracene from urban dust (SRM 1649a) and indeno[1,2,3-*cd*]fluoranthene from the wood smoke particulate sample were more susceptible to the storage condition and matrix type. All the PAH concentrations are listed together with the previously reported data in Tables S4, S5 and S6.

## Conclusion

We developed an automated 2D-LC/2D-GC system with two MSDs based on the previous system with two FIDs to improve the detectability and selectivity of PAHs in complex environmental matrices. The detector change involved the use of helium as the carrier gas, resulting in decreased column efficiency. Hence the method translation involved not only the carrier gas but also the column dimension to improve the separation. Two SRMs from the NIST, urban dust (SRM 1649a) and diesel particulate extract (SRM 1975), and a wood smoke particulate sample were used to validate the system developed. The results showed good correlation with those reported previously, especially greater agreement with the values obtained with the previous 2D system and from the NIST than those obtained by 1D-LC–GC/MS. The crude extracts originally prepared in the previous study and stored at -20 °C until analysis showed acceptable stability except for anthracene and indeno[1,2,3-*cd*]fluoranthene, possibly due to their chemical stability or the influence of the matrix during storage.

The 2D system developed enhanced the validity of the previous system and has the potential to be further developed by introduction of novel stationary phases in the second dimension.

## Electronic supplementary material

Below is the link to the electronic supplementary material.

ESM 1 (PDF 266 kb)
