# Supplementary material for: Automated clean-up, separation and detection of polycyclic aromatic hydrocarbons in particulate matter extracts using a 2D-LC/2D-GC system: a method translation from two FIDs to two MS detectors
Source: Anal Bioanal Chem. 2017 Jul 24;409(24):5619–29. doi: 10.1007/s00216-017-0509-1 (PMC5583270; doi:10.1007/s00216-017-0509-1)
Supplement: Supplementary file 1 — (PDF 266 kb) [file 216_2017_509_MOESM1_ESM.pdf]

Analytical and Bioanalytical Chemistry

Electronic Supplementary Material

Automated clean-up, separation and detection of polycyclic aromatic hydrocarbons in particulate matter extracts using a 2D-LC/2D-GC system: a method translation from two FIDs to two MS detectors

Hwanmi Lim, Trifa M. Ahmed, Christoffer Bergvall, Roger Westerholm

Table S1 List of PAHs and internal standards with CAS registry number and abbreviation

**PAH**	**CAS registry number**	**Abbreviation**
Dibenzothiophene	132-65-0	DBT
Phenanthrene	85-01-8	Phe
Anthracene	120-12-7	Ant/A
3-Methylphenanthrene	832-71-3	3-MPhe
2-Methylphenanthrene	2531-84-2	2-MPhe
2-Methylanthracene	613-12-7	2-MAnt
9-Methylphenanthrene	883-20-5	9-MPhe
1-Methylphenanthrene	832-69-9	1-MPhe
4*H*-Cyclopenta[*def*]phenanthrene	203-64-5	4*H*-CPP
3,6-Dimethylphenanthrene	1576-67-6	3,6-DMPhe
9-Methylanthracene	779-02-2	9-MAnt
3,9-Dimethylphenanthrene	66291-32-5	3,9-DMPhe
Fluoranthene	206-44-0	Flu/F
Pyrene	129-00-0	Pyr/P
1-Methylfluoranthene	25889-60-5	1-MFlu
11*H*-Benzo[*a*]fluorene	238-84-6	B[*a*]f
11*H*-Benzo[*b*]fluorene	243-17-4	B[*b*]f
2-Methylpyrene	3442-78-2	2-MPyr
4-Methylpyrene	3353-12-6	4-MPyr
1-Methylpyrene	2381-21-7	1-MPyr
Benzo[*c*]phenanthrene	195-19-7	B[*c*]Phe
Benzo[*ghi*]fluoranthene	203-12-3	B[*ghi*]F
Benzo[*b*]naphto[1,2-*d*]thiophene	205-43-6	B[*b*]NT
Benz[*a*]anthracene	56-55-3	B[*a*]A
Cyclopenta[*cd*]pyrene	27208-37-3	CPP
Chrysene	218-01-9	Chr
3-Methylchrysene	3351-31-3	3-MChr
2-Methylchrysene	3351-32-4	2-MChr
6-Methylchrysene	1705-85-7	6-MChr
1-Methylchrysene	3351-28-8	1-MChr
Benz[*e*]acephenanthrylene (Benzo[*b*]fluoranthene)	205-99-2	B[*b*]F
Benzo[*k*]fluoranthene	207-08-9	B[*k*]F
Benzo[*e*]pyrene	192-97-2	B[*e*]P
Benzo[*a*]pyrene	50-32-8	B[*a*]P
Perylene	198-55-0	Per/p
Indeno[1,2,3-*cd*]fluoranthene	193-43-1	I[1,2,3-*cd*]F
Indeno[1,2,3-*cd*]pyrene	193-39-5	I[1,2,3-*cd*]P
Dibenz[*a,h*]anthracene	53-70-3	DB[*a,h*]A
Benzo[*ghi*]perylene	191-24-2	B[*ghi*]p
Picene	213-46-7	Pic
Dibenzo[*def,p*]chrysene (Dibenzo[*a,l*]pyrene)	191-30-0	DB[*a,l*]P
Naphtho[1,2,3,4-*def*]chrysene (Dibenzo[*a,e*]pyrene)	192-65-4	DB[*a,e*]P
Coronene	191-07-1	Cor
Benzo[*rst*]pentaphene (Dibenzo[*a,i*]pyrene)	189-55-9	DB[*a,i*]P
Dibenzo[*b,def*]chrysene (Dibenzo[*a,h*]pyrene)	189-64-0	DB[*a,h*]P
**Internal standards**	**CAS registry number**	**Abbreviation**
Phenanthrene-d_{10}	1517-22-2	Phe-d_{10}
Pyrene-d_{10}	1718-52-1	Pyr-d_{10}
Benz[*a*]anthracene-d_{12}	1718-53-2	B[*a*]A-d_{12}
Benz[*a*]pyrene-d_{12}	63466-71-7	B[*a*]P-d_{12}
Benzo[*ghi*]perylrene-d_{12}	93951-66-7	B[*ghi*]p-d_{12}
Coronene-d_{12}	16083-32-2	Cor-d_{12}
Benzo[*rst*]pentaphene-d_{14} (Dibenzo[*a,i*]pyrene-d_{14})	158776-07-9	DB[*a,i*]P-d_{14}

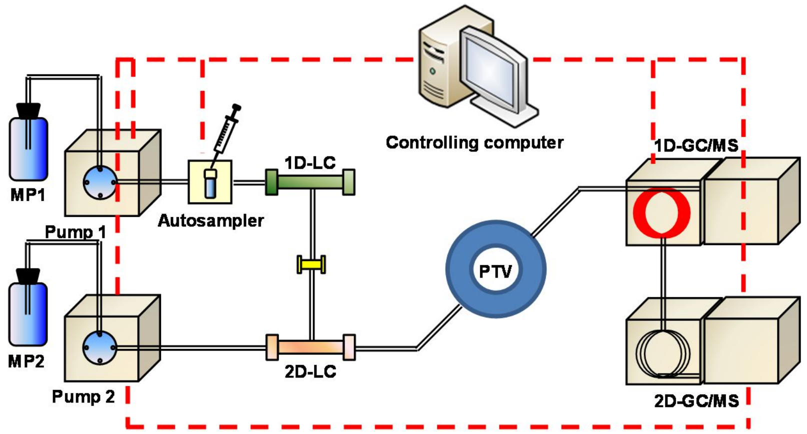

Fig. S1 Scheme of 2D-LC/2D-GC/MS system

MP: mobile phase, 1D: first dimension, 2D: second dimension, LC: liquid chromatography, PTV: programmable temperature vaporizer, GC/MS: gas chromatography/mass spectrometry

Table S2 GC/MS parameters used in 2D-LC/2D-GC/MS system

**Injection**		PTV injection in solvent vent mode<br>50 °C until the sample introduction (18.9 min), 700 °C/min, 400 °C (3 min), 100 °C/min, 300 °C
**Carrier gas**		Helium
**1D-GC**	Flow	Constant flow, 0.875 mL/min
	Column	LTM DB-17MS 15 m × 0.25 mm, 0.15 µm phase
	Oven	40 °C (20.9 min), 40 °C/min, 180 °C, 2 °C/min, 214 °C (36 min), 20 °C/min, 250 °C (3 min), 5 °C/min, 320 °C (9 min)
	Detection	Triple Quad MS, EI in SIM, source 230 °C, transfer line 250 °C
**2D-GC**	Flow	Constant flow, 0.875 mL/min
	Column	LC50 5 m × 0.25 mm, 0.10 µm phase
	Oven	40 °C (20.9 min), 40 °C/min, 190 °C (8 min), 120 °C/min, 260 °C (72 min)
	Detection	Quad MS, EI in SIM, source 300 °C, MS Quad 150 °C, transfer line 250 °C

Table S3 Deans switch valve programme for the 2D-LC/2D-GC/MS system

**Time (min)**	**Time (min)**[a]	**Valve**
0.0	0.0	on
34.5	34.3	off
38.0	37.8	on
54.0	53.8	off
80.5	80.3	on
96.0	95.8	off

[a] Adjusted time obtained by subtracting 0.2 minutes from the original retention time to compensate the switching valve's time delay

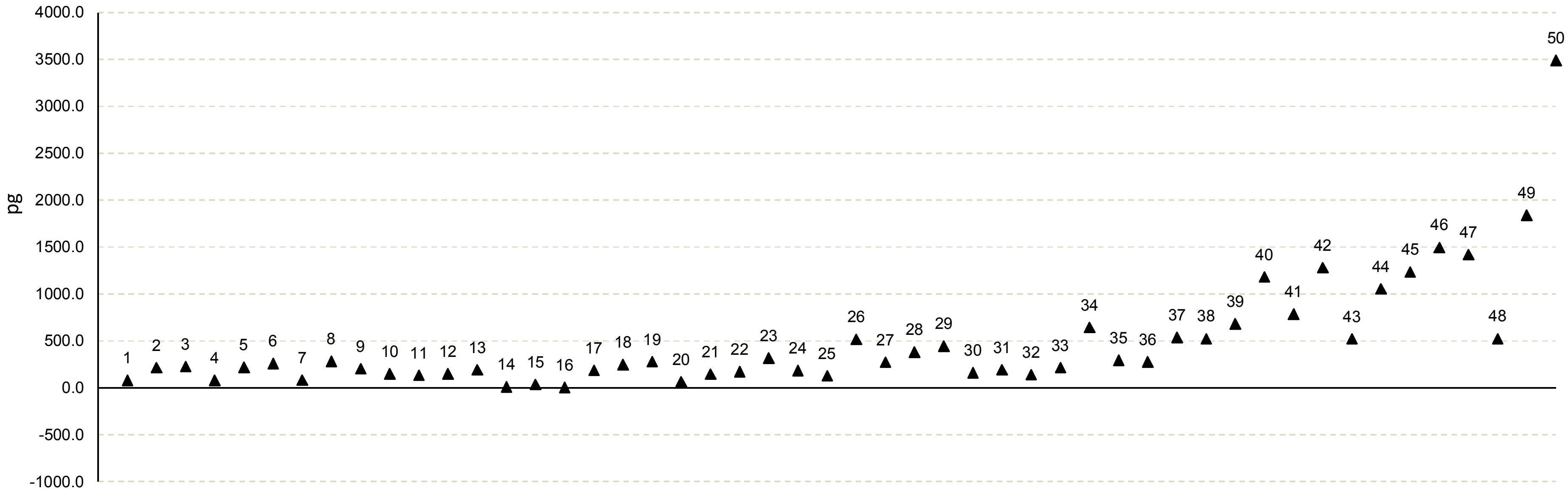

Fig. S2 Scatter plot showing LOQ of each PAH and internal standard used in the present study

1: DBT, 2: Phe-d_{10}, 3: Phe, 4: Ant, 5: 3-MPhe, 6: 2-MPhe, 7: 2-MAnt, 8: 9-MPhe, 9: 1-MPhe, 10: 4*H*-CPP, 11: 3,6-DMPhe, 12: 9-MAnt, 13: 3,9-DMPhe, 14: Flu, 15: Pyr-d_{10}, 16: Pyr, 17: 1-MFlu, 18: B[*a*]f, 19: B[*b*]f, 20: 2-MPyr, 21: 4-MPyr, 22: 1-MPyr, 23: B[*c*]Phe, 24: B[*ghi*]F, 25: B[*b*]NT, 26: B[*a*]A-d_{12}, 27: B[*a*]A, 28: CPP, 29: Chr, 30: 3-MChr, 31: 2-MChr, 32: 6-MChr, 33: 1-MChr, 34: B[*b*]F, 35: B[*k*]F, 36: B[*e*]P, 37: B[*a*]P-d_{12}, 38: B[*a*]P, 39: Per, 40: I[1,2,3-*cd*]F, 41: I[1,2,3-*cd*]P, 42: DB[*a,h*]A, 43: B[*ghi*]p-d12, 44: B[*ghi*]p, 45: Pic 46: DB[*a,l*]P, 47: DB[*a,e*]P 48: Cor-d_{12}, 49: Cor, , 50: DB[*a,i*]P+DB[*a,h*]P. All abbreviations are listed in Table S1.

Table S4 PAH concentrations (ng/mg) from SRM 1649a (urban dust) compared with those reported previously and certified values from the NIST

**SRM 1649a**	**This study**[a]	**Ahmed et al., 2013**[b] **[1]**	**Ahmed et al., 2013**[c] **[1]**	**NIST CoA, 2007 (1649a) [2]**	**NIST CoA, 2016 (1649b) [3]**
Phe	4.62 ± 0.22	5.19 ± 0.0.3	5.20 ± 0.38	4.14 ± 0.37	4.40 ± 0.02[d]
Ant	0.709 ± 0.035	0.747 ± 0.036	0.862 ± 0.049	0.432 ± 0.082	0.978 ± 0.021[d]
Flu	6.09 ± 0.12	7.50 ± 0.66	6.44 ± 0.03	6.45 ± 0.18	6.60 ± 0.04[d]
Pyr	5.06 ± 0.02	6.18 ± 0.11	5.48 ± 0.03	5.29 ± 0.25	4.98 ± 0.14
B[*a*]A	2.43 ± 0.06	2.19 ± 0.08	2.52 ± 0.06	2.208 ± 0.073	2.35 ± 0.12[d]
Chr	3.48 ± 0.22	3.10 ± 0.13	3.50 ± 0.11	3.049 ± 0.060	3.045 ± 0.028
B[*b*]F	5.93 ± 0.17	5.82 ± 0.33	5.80 ± 0.07	6.45 ± 0.64	6.18 ± 0.18
B[*k*]F	1.81 ± 0.16	1.96 ± 0.10	1.81 ± 0.01	1.913 ± 0.031	1.702 ± 0.049
B[*e*]P	3.40 ± 0.18	3.30 ± 0.33	3.53 ± 0.05	3.09 ± 0.19	2.974 ± 0.053
B[*a*]P	2.64 (SD<0.01)	2.55 ± 0.01	2.69 ± 0.06	2.509 ± 0.087	3.04 ± 0.10[d]
Per	0.650 ± 0.042	2.66 ± 0.17	0.700 ± 0.024	0.646 ± 0.075	0.614 ± 0.011
I[1,2,3-*cd*]P	3.30 ± 0.10	3.90 ± 0.17	2.16 ± 0.02	3.18 ± 0.72	2.89 ± 0.16
DB[*a,h*]A	0.326 ± 0.023	N/A[e]	0.394 ± 0.003	0.288 ± 0.023	N/A[e]
B[*ghi*]p	3.92 ± 0.18	3.58 ± 0.09	4.22 ± 0.07	4.01 ± 0.91	4.31 ± 0.08[d]
Pic	0.481 ± 0.028	0.608 ± 0.032	0.415 ± 0.019	0.426 ± 0.022	0.399 ± 0.022
DB[*a,e*]P	0.512 ± 0.065	N/A[e]	0.520 ± 0.008	0.565 ± 0.060	0.567 ± 0.025

[a] 2D-LC/2D-GC/MS (*n*=3)

[b] 2D-LC/2D-GC/FID (*n*=3)

[c] LC-GC/MS (*n*=2)

[d] Reference mass fraction values based on the pressurised fluid extraction at 200 °C

[e] Data not available

Table S5 PAH concentrations (ng/mg) from SRM 1975 (diesel particulate extract) compared with those reported previously and certified values from the NIST

**SRM 1975**	**This study[a]**	**Ahmed et al., 2013[b] [1]**	**Ahmed et al., 2013[c] [1]**	**NIST CoA, 2008 (1975) [4]**
Phe	8.61 ± 0.49	9.11 ± 0.34	8.90 ± 0.15	8.00 ± 0.20
Flu	15.3 ± 0.03	13.6 ± 0.7	12.5 (SD<0.1)	13.5 ± 0.6
B[*a*]A	0.0772 ± 0.0028	0.0952 ± 0.0162	0.0724 ± 0.0026	0.092 ± 0.015
Chr	1.98 ± 0.19	2.02 ± 0.13	2.51 ± 0.34	1.95 ± 0.07
B[*b*]F	3.59 ± 0.07	3.40 ± 0.08	3.42 ± 0.02	3.20 ± 0.10
B[*k*]F	0.176 ± 0.013	0.166 ± 0.011	0.175 ± 0.025	0.174 ± 0.050
B[*e*]P	0.294 ± 0.014	0.280 ± 0.004	0.289 ± 0.007	0.268 ± 0.023

[a] 2D-LC/2D-GC/MS (*n*=3)

[b] 2D-LC/2D-GC/FID (*n*=3)

[c] LC-GC/MS (*n*=2)

Table S6 PAH concentrations (ng/mg) from wood smoke particulates compared with the values from references

**PAH**	**This study[a]**	**Ahmed et al., 2013[b] [1]**	**Bølling et al., 2012[c] [5]**	**PAH**	**This study[a]**	**Ahmed et al., 2013[b] [1]**	**Bølling et al., 2012[c] [5]**
DBT	<LOQ[d]	N/A[e]	N/A[e]	B[*b*]NT	<LOQ[d]	N/A[e]	0.537 ± 0.053
Phe	366 ± 6	403 ± 3	377 ± 6	B[*a*]A	717 ± 35	710 ± 60	711 ± 3
Ant	42.8 ± 0.8	45.3 ± 0.8	45.1 ± 1.8	CPP	173 ± 9	171 ± 20	636 ± 76
3-MPhe	40.8 ± 1.4	40.0 ± 5.7	44.6 ± 3.3	Chr	861 ± 19	877 ± 65	856 ± 75
2-MPhe	45.2 ± 1.5	52.8 ± 6.1	55.2 ± 3.5	3-MChr	30.9 ± 4.4	N/A[e]	28.9 ± 0.5
2-MAnt	10.7 ± 0.4	11.7 ± 1.8	11.3 ± 0.6	2-MChr	40.0 ± 3.7	N/A[e]	45.2 ± 0.8
9-MPhe	27.6 ± 2.9	27.3 ± 2.1	33.5 ± 3.0	6-MChr	27.5 ± 0.4	N/A[e]	34.3 ± 0.3
1-MPhe	52.7 ± 0.6	59.2 ± 7.5	65.1 ± 4.3	1-MChr	35.7 ± 1.9	58.9 ± 2.9	36.0 ± 0.4
4*H*-CPP	86.0 ± 3.1	77.1 ± 3.9	83.6 ± 6.4	B[*b*]F	707 ± 65	625 ± 16	681 ± 5
3,6-DMPhe	5.60 ± 0.33	4.99 ± 0.18	3.06 ± 0.27	B[*k*]F	340 ± 50	325 ± 15	338 ± 2
9-MAnt	13.6 ± 0.6	11.3 ± 0.1	14.4 ± 1.1	B[*e*]P	448 ± 51	501 ± 4	499 ± 1
3,9-DMPhe	9.26 ± 0.57	9.58 ± 0.32	8.14 ± 0.14	B[*a*]P	615 ± 22	553 ± 3	640 ± 6
Flu	1630 ± 70	1670 ± 68	1660 ± 10	Per	107 ± 11	612 ± 33	98.1 ± 0.1
Pyr	1740 ± 20	1490 ± 41	1740 ± 20	I[1,2,3-*cd*]F	223 ± 10	59.8 ± 6.7	56.0 ± 0.2
1-MFlu	140 ± 1	132 ± 3	162 ± 5	I[1,2,3-*cd*]P	529 ± 31	586 ± 17	420 ± 1
B[*a*]f	118 ± 6	125 ± 18	106 ± 4	DB[*a,h*]A	56.8 ± 3.3	69.3 ± 7.7	62.8 ± 1.3
B[*b*]f	53.3 ± 5.6	57.2 ± 1.7	51.8 ± 1.4	B[*ghi*]p	479 ± 50	527 ± 10	562 ± 2
2-MPyr	77.2 ± 6.7	61.8 ± 1.1	62.6 ± 1.5	Pic	84.2 ± 5.3	103 ± 29	72.0 ± 1.3
4-MPyr	89.9 ± 2.2	96.2 ± 11.3	97.1 ± 2.7	DB[*a,l*]P	43.5 ± 5.8	N/A[e]	N/A[e]
1-MPyr	106 ± 4	99.2 ± 7.6	113 ± 3	DB[*a,e*]P	646 ± 80	N/A[e]	N/A[e]
B[*c*]Phe	204 ± 6	218 ± 18	232 ± 3	Cor	322 ± 5	747 ± 178	268 ± 1
B[*ghi*]F	515 ± 7	623 ± 74	621 ± 6				

[a] 2D-LC/2D-GC/MS (*n*=3)

[b] 2D-LC/2D-GC/FID (*n*=3)

[c] LC-GC/MS (*n*=2)

[d] Below LOQ

[e] Data not available

References

1. Ahmed TM, Lim H, Bergvall C, Westerholm R. Automated clean-up, separation and detection of polycyclic aromatic hydrocarbons in particulate matter extracts from urban dust and diesel standard reference materials using a 2D-LC/2D-GC system. Anal Bioanal Chem. 2013;405:8215-22.
2. NIST. Certificate of analysis: Standard reference material 1649a urban dust. National Institute of Standards and Technology. 2007.
3. NIST. Certificate of analysis: Standard Reference Material 1649b urban dust. National Institute of Standards and Technology. 2016.
4. NIST. Certificate of analysis: Standard Reference Material 1975 diesel particulate extract. National Institute of Standards and Technology. 2008.
5. Bølling AK, Totlandsdal AI, Sallsten G, Braun A, Westerholm R, Bergvall C, Boman J, Dahlman HJ, Sehlstedt M, Cassee F, Sandstrom T, Schwarze PE, Herseth JI. Wood smoke particles from different combustion phases induce similar pro-inflammatory effects in a co-culture of monocyte and pneumocyte cell lines. Part Fibre Toxicol. 2012;9:45.
